# F40. NEUREXIN-1α (NRNX1α) HYPOFUNCTION INDUCES SCHIZOPHRENIA-RELEVANT DEFICITS IN CEREBRAL METABOLISM, COGNITIVE PROCESSING SPEED AND COGNITIVE FLEXIBILITY

**DOI:** 10.1093/schbul/sby017.571

**Published:** 2018-04-01

**Authors:** Rebecca Hughes, Jayde Whittingham-Dowd, Greg Bristow, Stephen Clapcote, Susan Broughton, Neil Dawson

**Affiliations:** 1 Lancaster University; 2 University of Leeds

## Abstract

**Background:**

Heterozygous deletions in NEUREXIN-1 (NRXN1) substantially increase the risk of developing schizophrenia (SZ) (Rujescu et al., 2009. Hum Mol Genet 18(5):988–96). We currently have little understanding into the mechanisms by which NRXN1 impacts on the brain to increase the risk of developing the disorder, and to which symptom domains NRXN1 may contribute. Patients with schizophrenia show deficits in cognitive processing speed and cognitive flexibility (Sanchez et al., 2009. J Clin Psychiat. 70(6):888–896, Dieci et al., 1997. Schizophr Res. 25(1):33–42). In addition, patients show characteristic alterations in brain function including “hypofrontality”; prefrontal cortex hypometabolism (Hill et al., 2004. Acta Psychiatr Scand. 110(4):243–56). Here we characterise, in a transgenic mouse model, the impact of Nrxn1α hypofunction on cognitive flexibility, processing speed and cerebral metabolism to determine the potential translational relevance of the model to SZ.

**Methods:**

Nrxn1α heterozygous (Hz) mice and their wild-type (Wt) littermates, of both sexes, were tested at 3, 6, 9 and 12 months old using a between-groups design. Associative learning and cognitive flexibility (reversal learning and set shifting) were assessed in a two choice odour based set shifting task (adapted from Young et al., 2010. Cog Affect Behav Neurosci. 10(2):243–251). Mice completed a series of testing phases that included two odour discrimination phases (OD1 and OD2), one reversal learning phase (OD2R) and an extra-dimensional shift (EDS), with animals shifting attentional set from odour to location. The criterion to successfully complete each phase was set at 6 consecutive correct choices. The number of trials to reach criterion, percentage correct and average latency for correct choices were recorded. After behavioural testing cerebral metabolism was determined in 49 brain regions using 14C-2-deoxyglucose functional brain imaging (Dawson et al., 2015. Transl Psychiatry. 5(5):e569). Data were analysed using ANOVA and t-test with Bonferroni correction. Significance was set at p<0.05.

**Results:**

In the associative learning phases of the task (OD1 and OD2) Nrxn1α Hz mice took a similar number of trials as Wt controls to reach criteria. Nrxn1α Hz mice also completed a similar percentage of correct trials during these phases. This suggests that associative learning is not impaired in Nrxn1α Hz mice. However, Nrxn1α Hz mice showed a significant increase in the latency of correct choices in comparison to Wt animals during these phases, supporting significantly decreased processing speed in these animals. We also found that reversal learning (CDR2) was impaired in Nrxn1α Hz mice, evidenced by a significant increase in trials to criterion relative to Wt controls. In the brain imaging study, we found that Nrxn1α Hz mice show significant hypofrontality, with a reduced rate of metabolism in the anterior and medial prelimbic cortex (aPrL, mPrL). By contrast, Nrxn1α heterozygous mice show significant hypermetabolism in the dorsal raphe (DR), ventral tegmental area (VTA) and retrosplenial cortex (RSC).

**Discussion:**

Nrxn1α heterozygosity induces SZ-like impairments in cognitive processing speed, cognitive flexibility (reversal learning) and cerebral metabolism, including the induction of hypofrontality. Nrxn1α heterozygosity also alters metabolism in neuromodulatory brain regions, including the serotonergic DR and dopaminergic VTA, which may also contribute to its impact on cognition. These data give new insight into the mechanisms by which NRXN1 heterozygosity increases the risk of developing SZ and suggest that Nrxn1α Hz mice provide a translational tool for drug discovery in relation to the cognitive deficits seen in the disorder.

